# Main causes of water-electrolyte disturbances in patients with acute brain injury: central diabetes insipidus and cerebral salt wasting syndrome

**DOI:** 10.1186/cc13624

**Published:** 2014-03-17

**Authors:** L Tsentsiper, N Dryagina

**Affiliations:** 1 Russian Polenov's Neurosurgical Institute, Saint Petersburg, Russia

## Introduction

Water-electrolyte disturbances are one of the most common complications of acute brain injury of various origins, threatening the life of the patient and requiring timely correction. In this work we studied the structure of water-electrolyte complications in patients in the neurological intensive care with acute brain injury.

## Methods

We analyzed 259 cases of water-electrolyte disturbances that developed in patients treated in the Department of Intensive Care of Russian Polenov's Neurosurgical Institute from 2001 to 2012. Patients were between 16 and 55 years old. A total of 142 patients were operated for brain tumor, 72 of them of basal-supratentorial localization; eight severe brain trauma; 62 of the hemorrhagic type of stroke, one herpes encephalitis. We excluded from this study the patients with heart and renal failure receiving diuretics. We measured BP, HR, CVP, hourly and daily urine output, level of K and Na in plasma, brain natriuretic peptide (BNP) one to four times a day, and levels of K and Na in urine in single and daily servings. All patients were receiving dexamethasone at a dose between 8 and 32 mg/day as an anti-edema therapy, and thus levels of ACTH and cortisol were not investigated.

## Results

Central diabetes insipidus (CDI) developed in 106 patients. Against the background of substitution therapy, we did not observe the development of complications, hypovolemia, hypernatremia, or hyperosmolality. Out of 48 cases of CSWS, in 12 cases the symptoms interleaved with the symptoms of CDI. For 24 patients, CSWS was complicated by depressed consciousness, for three patients by convulsions against the background of hypovolemia, hyponatremia, or hypo-osmolality. No correlation between BPN level and absence of CSWS symptoms was found.

## Conclusion

In patients with acute brain trauma we observed two syndromes causing water-electrolyte disturbance - CDI and CSWS. CSWS generally is more severe than CDI. We could not determine a correlation between BPN level and the absence of presence of the CSWS symptoms.

